# Synchronous NET and colorectal cancer development: a case report

**DOI:** 10.1186/s40792-020-0777-4

**Published:** 2020-01-09

**Authors:** Shinsei Yumoto, Yuji Miyamoto, Takahiko Akiyama, Yuki Kiyozumi, Kojiro Eto, Yukiharu Hiyoshi, Yohei Nagai, Masaaki Iwatsuki, Yoshifumi Baba, Shiro Iwagami, Naoya Yoshida, Hideo Baba

**Affiliations:** Department of Gastroenterological Surgery, Graduate School of Medical Sciences, Kumamoto University, 1-1-1 Honjo, Chuo-ku, Kumamoto, 860-8556 Japan

**Keywords:** NET, Colorectal cancer, Everolimus

## Abstract

**Background:**

The incidence of synchronous gastrointestinal neuroendocrine tumors (GI-NETs) and colorectal cancer is very low.

**Case presentation:**

We present a 72-year-old man diagnosed with a rectal neuroendocrine tumor (NET) with multiple organ metastases and simultaneous sigmoid colon cancer. Although the NET was his prognostic factor, he underwent a laparoscopic sigmoidectomy at first because it was expected that the colon cancer would cause obstruction or bleeding during NET treatment. Subsequently, he started taking everolimus.

**Conclusions:**

We should consider surgical resection of the synchronous cancer before systemic therapy for a GI-NET regardless of the difference in prognosis between synchronous tumors, if the cancer may impair the continuation of systemic therapy.

## Background

Neuroendocrine tumors (NETs) are relatively rare, but are frequently associated with synchronous or metachronous secondary primary malignancies [1]. Although a few cases of synchronous colorectal cancer with gastrointestinal neuroendocrine tumors (GI-NETs) have been reported [2–4], to the best of our knowledge there are no cases in which a surgical resection was performed for cancer and chemotherapy for a GI-NET with multiple organ metastases. Here, we report a rare case where the patient was diagnosed with synchronous colorectal cancer and a rectal NET and underwent colectomy and chemotherapy.

## Case presentation

**Fig. 1:**
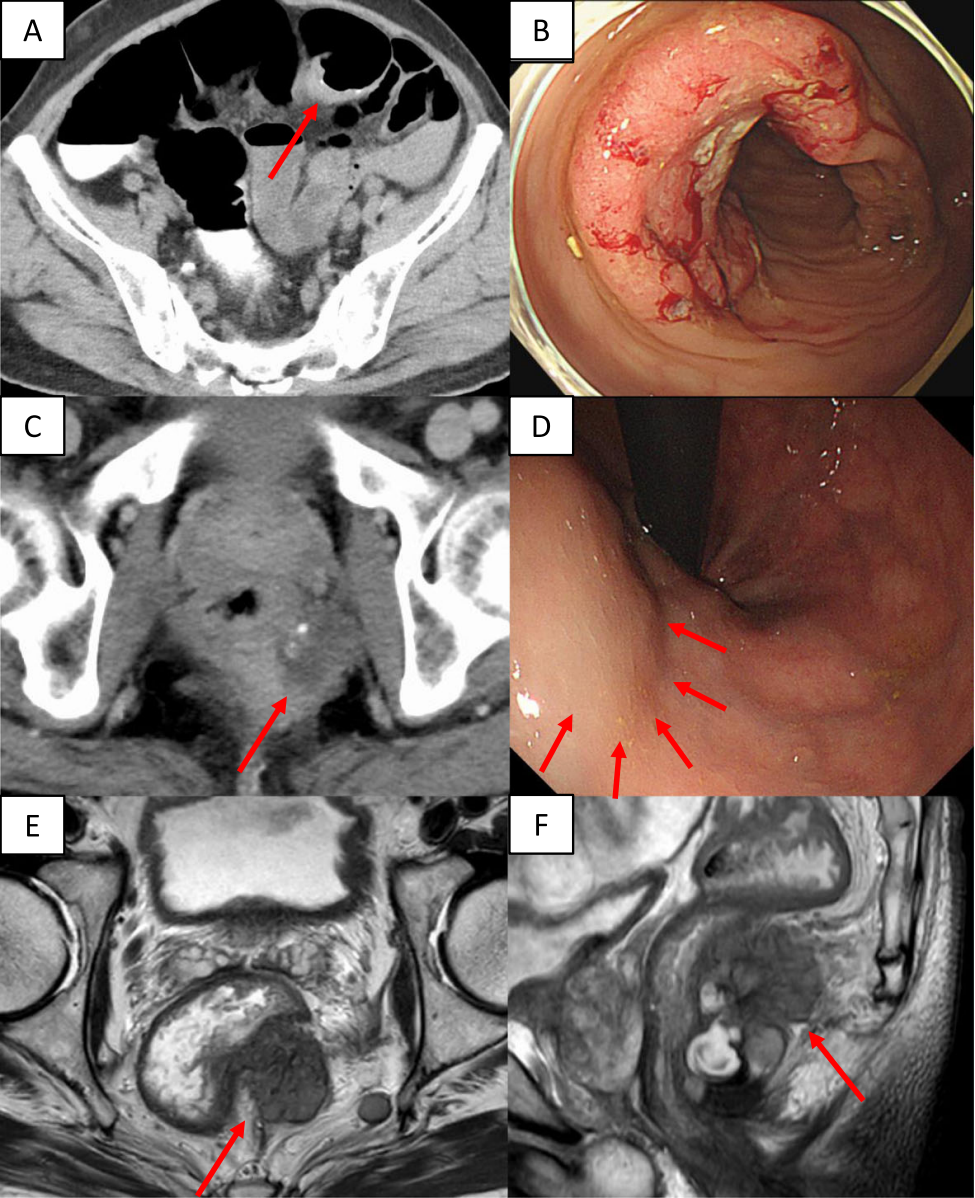
Image of the sigmoid colon cancer and rectal NET. A contrast-enhanced CT (**a**) and a colonoscopy (**b**) showed a sigmoid colon cancer. A contrast-enhanced CT (**c**) and a colonoscopy (**d**) showed a rectal NET. An MRI (**e** axial views, **f** sagittal views) showed the rectal tumor definitely developed from the rectum. Each arrow shows the lesion

**Fig. 2:**
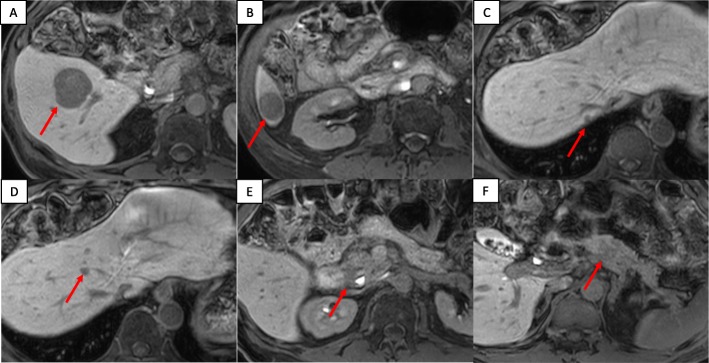
MRI image of liver and pancreatic tumors. An abdominal MRI showed multiple liver and pancreatic tumors. Each arrow shows the lesion. **a** S5 tumor. **b** S6 tumor. **c** S7 tumor. **d** S8 tumor. **e** Pancreatic head tumor. **f** Pancreatic body tumor

**Fig. 3:**
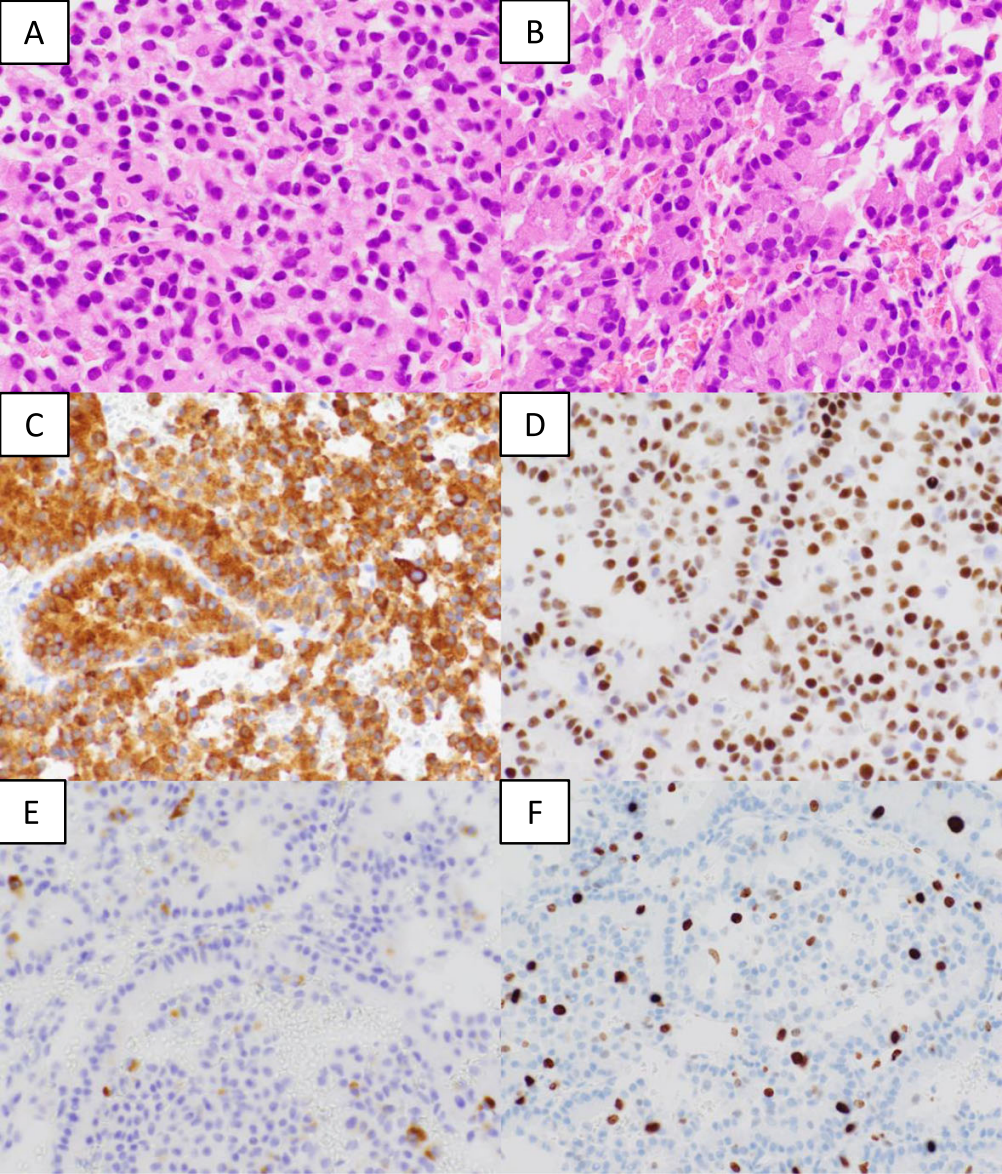
Pathological findings of rectum and liver tumors. Both tumors displayed as NETG2s. **a** Rectal tumor (hematoxylin and eosin staining). **b** Liver tumor (hematoxylin and eosin staining). **c** Synaptophysin positive. **d** INSM1 positive. **e** Chromogranin A positive. **f** MIB-1/Ki 67 positive cells was about 10%. **c**–**f** Specimens of the rectum

**Fig. 4:**
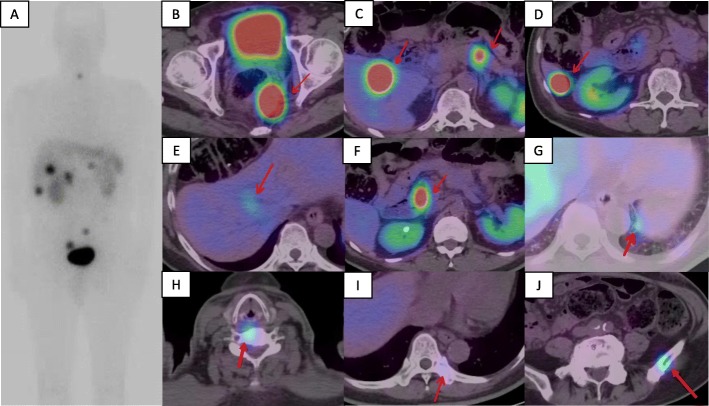
Whole body Octreoscan image. An Octreoscan showed abnormal uptake in multiple organs. Each arrow shows the lesion. **a** A whole-body planar image (anterior view). **b** Rectum (primary site). **c** S5 liver tumor and pancreatic body tumor. **d** S6 liver tumor. **e** S8 liver tumor. **f** Pancreatic head tumor. **g** Left lung bases. **h** Sixth cervical vertebra. **i** Ninth thoracic vertebra. **j** Left ilium

**Fig. 5:**
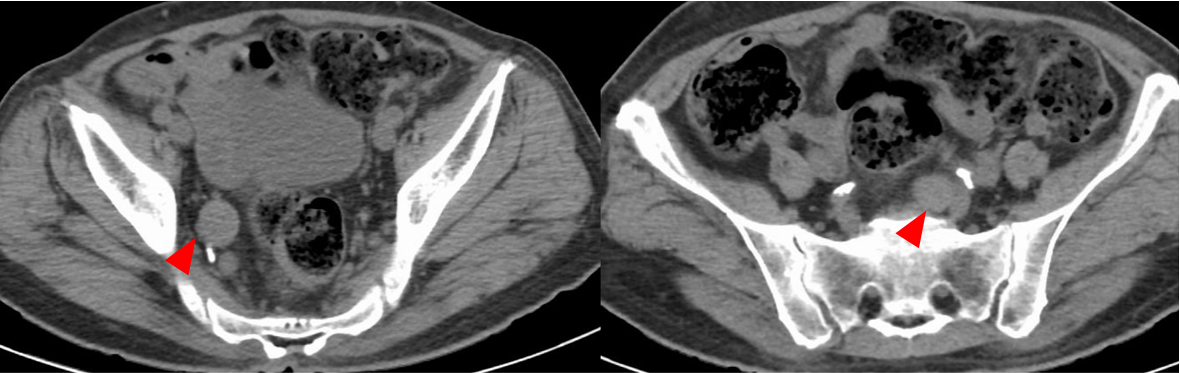
CT image of the lymphadenopathy around the rectum. Arrow heads show the lymphadenopathy around the rectum

A 72-year-old man presented at our hospital to undergo an additional examination for the multiple liver tumors found in his periodic abdominal ultrasonography. His medical history included type 2 diabetes mellitus treated with injected insulin. Contrast-enhanced computed tomography (CT) showed multiple liver and pancreatic tumors, with neoplastic lesions in the sigmoid colon and the dorsum of the rectum (Fig. 1a, c). A colonoscopy revealed a type 2 lesion at the sigmoid colon, and the biopsy showed an adenocarcinoma (Fig. 1b). In addition, a submucosal tumor was identified in the rectum (Fig. 1d). The tumor size was 45 mm. A magnetic resonance imaging (MRI) showed an extramural growth-type submucosal tumor developing from the rectum (Fig. 1e, f). An MRI scan also showed multiple liver and pancreatic tumors, but their image patterns were atypical of metastases of colon cancer (Fig. 2). We therefore performed a CT-guided biopsy (CTGB) for the liver tumors and the rectal dorsal tumor. Both biopsy results showed tumor cells with a rosette-like sequence consisting of synaptophysin (+), INSM1 (+), chromogranin A (+), β-catenin (the cell membrane positive, the nucleus negative), CK7 (−), CK20 (−), CDX2 (−), CD56 (−), CD10 (−), and trypsin (−) in immunostaining. Mitotic index was less than 2 per 10 high-power fields (HPF), and Ki-67 index was about 10% (Fig. 3). Also, the pathological specimens revealed neither lymphatic nor venous invasion. Furthermore, an Octreoscan revealed metastases of the liver, pancreas, lungs, ilium, and spine (Fig. 4). In view of the conspicuous lymphadenopathy in the pelvic region (Fig. 5), we considered that the primary site of the NET was the rectum. From the above, our final diagnosis was a rectal NETG2 (cT3N1M1c cStage IV, Union for International Cancer Control [UICC] 8th edition) and simultaneous sigmoid colon cancer (cT3N0M0 cStage II, UICC 8th edition).

First, we performed a laparoscopic sigmoidectomy (D3 dissection) because the sigmoid colon cancer was at risk of obstruction and bleeding. The postoperative course was good, and the patient was discharged on postoperative day 8. The histological examination of the resected specimen revealed adenocarcinoma, tubular, moderately differentiated and pT3, pN0. Thereafter, he started taking 10 mg per day of everolimus on postoperative day 51. Currently, 2 months have passed since he started the treatment, which is continuing without the cancer worsening.

## Discussion

NETs are relatively rare tumors and located most commonly in the gastrointestinal tract (55%) and bronchopulmonary system (30%) according to a report from the SEER database [5]. Within the gastrointestinal tract, the rectum is the second most common site (20%) after the small intestine [6], and rectal NETs are estimated to occur at a rate of 0.14 to 0.76/100,000 cases [7, 8]. They are typically nonfunctioning and asymptomatic, and thus extremely difficult to diagnose. Recently, however, due to the widespread implementation of colonoscopy as a screening tool, the rate of detection of colorectal NETs is increasing [9, 10]. Most cases of rectal NETs (80–88%) are localized, whereas the remaining NETs (12–20%) are diagnosed with regional lymph node spread and/or distant metastases (21% G1, 30% G2, and 50% neuroendocrine carcinomas [NECs]) [11].

In our case, the patient had no symptoms, and multiple liver tumors were incidentally discovered by abdominal ultrasonography. A CT and a colonoscopy for systemic examination resulted in an accurate diagnosis, and a CT-guided biopsy (CTGB) of the liver and rectum confirmed a NETG2. The liver biopsy was significant in considering whether the liver tumors were metastases of the NET or of the simultaneously discovered colon cancer. The Octreoscan showed an abnormal uptake in multiple organs including the pancreas, and it was difficult to determine the primary site. Finally, clinically, we considered the primary site to be the rectum in terms of the multiplicity of the pancreatic tumors, MRI image pattern concordance between the liver and the pancreatic tumors, and the bulky lymphadenopathy around the rectum. However, we could not unconditionally rule out the possibility of synchronous tumors of the rectal NET and a pancreatic neuroendocrine tumor (P-NET) with either liver metastases of the NET or rectal and liver metastases of the P-NET. According to Yao et al., in patients with an advanced, progressive, nonfunctional GI-NET, treatment with everolimus was associated with statistically significant and clinically meaningful prolongation of progression-free survival (PFS) [12]. Thus, we immediately started the patient on 10 mg per day of everolimus after the surgery.

This case emphasizes that for synchronous cancer, if the cancer can impede continued systemic therapy, we should consider surgical resection before systemic therapy for the NET regardless of the difference in prognosis between the synchronous tumors. Of course, palliative or cytoreductive surgery for the NET should be considered at the same time, regardless of whether it is a primary or metastatic lesion. This is even more true if the NET involves local symptoms. However, in patients diagnosed with a NET with distant metastasis, early systemic therapy is extremely important. We should take not only the patient’s general condition, but also their condition after surgery into consideration in determining the indications for surgery or surgical procedures.

Generally, GI-NETs often show hematogenous metastases, and the most frequent sites of distant spread are the liver, bones, and peritoneal cavity [13–16]. According to some reports, the tumor size, depth of invasion, and lymph node involvement are all significant predictors of malignant behavior in rectal NETs [17]. Tumors smaller than 1 cm are rarely metastatic, with only 3.7% of rectal NETs that are 0.5 cm or smaller being metastatic at the time of diagnosis; rates of metastatic disease increase to 13.2% in tumors 0.5 to 1.0 cm in size, and to 26–28% in tumors 1 to 2 cm in size [18]. Up to 70% of tumors larger than 2 cm may be metastatic [14, 19]. In addition, many previous studies have reported that the presence of muscularis propria or lymphovascular invasion in a rectal NET is a strong risk factor for metastasis [20–22]. In our case, the tumor size was 45 mm and both CT and MRI indicated tumor invasion of the muscularis propria. Therefore, we concluded that these are the reasons why multiple organ metastases developed, despite the rectal NET being well-differentiated (G2). This also supported the diagnosis of a rectal primary NET. Interestingly, the pancreas is extremely rare as a distant metastasis site of a NET [23, 24].

It has been reported in some of the English literature that NETs are associated with synchronous or metachronous secondary primary malignancies (SPMs): rates of SPMs are up to 55% in NETs [25], and Tichansky et al. reported that 8% of patients with colorectal carcinoid also had synchronous cancer [26]. Additionally, Winn et al. reported that in synchronous colon adenocarcinomas with a gastrointestinal carcinoid tumor, the most common location was the sigmoid colon [2]. There are several hypotheses to explain the relationship between NETs and SPMs, including the field-effect theory, the stem cell theory, the neuropeptides theory, the genetic defect hypothesis, and the immunodeficiency theory [25, 27–33]. When we found that our patient had a simultaneous sigmoid colon cancer, the rectal NET was already well-developed and had multiple organ metastases at the time of diagnosis. To the best of our knowledge, no cases of synchronous colorectal cancer and a GI-NET with distant metastasis have been reported. As the GI-NET may coexist with SPMs, the diagnosis of a GI-NET must be extensively evaluated for SPMs during the workup and follow-up periods. We recommend performing a CT and upper and lower endoscopies.

## Conclusions

This is the first report of a case of a GI-NET with synchronous colorectal cancer that involves multiple organ metastases. If it appears difficult for patients to continue future systemic therapy with their local symptoms of synchronous cancer, we should consider surgical resection of the synchronous cancer before systemic therapy for the GI-NET, regardless of the difference in prognosis between the synchronous tumors. Further studies are required to clarify the mechanisms of carcinogenesis associated with GI-NETs and synchronous tumors.

## Data Availability

All data generated or analyzed during this study are included in this published article.

## Abbreviations

CT: Computed tomography
CTGB: CT guided biopsy
EUS: Endoscopic ultrasound
GI-NET(s): Gastrointestinal neuroendocrine tumor(s)
HPF: High-power fields
MRI: Magnet resonance imaging
NECs: Neuroendocrine carcinomas
NET(s): Neuroendocrine tumor(s)
PFS: Progression-free survival
P-NET: Pancreatic neuroendocrine tumor
SPMs: Secondary primary malignancies
UICC: Union for International Cancer Control
